# Germicidal lamps using UV-C radiation may pose health safety issues: a biomolecular analysis of their effects on apoptosis and senescence

**DOI:** 10.18632/aging.205787

**Published:** 2024-05-02

**Authors:** Nicola Alessio, Alessia Ambrosino, Andrea Boggi, Domenico Aprile, Iole Pinto, Giovanni Galano, Umberto Galderisi, Giovanni Di Bernardo

**Affiliations:** 1 Biotechnology and Molecular Biology Section, Department of Experimental Medicine, School of Medicine, University of Campania Luigi Vanvitelli, Naples 80138, Italy; 2 Physical Agents Sector, Regional Public Health Laboratory, Siena 53100, Italy; 3 ASL Napoli 1 Centro P.S.I. Napoli Est-Barra, Naples 80147, Italy; 4 Center for Biotechnology, Sbarro Institute for Cancer Research and Molecular Medicine, Temple University, Philadelphia, PA 19122, USA

**Keywords:** senescence, apoptosis, UV light, public health

## Abstract

The battle against the COVID-19 pandemic has spurred a heightened state of vigilance in global healthcare, leading to the proliferation of diverse sanitization methods. Among these approaches, germicidal lamps utilizing ultraviolet (UV) rays, particularly UV-C (wavelength ranging from 280 to 100 nm), have gained prominence for domestic use. These light-emitting diode (LED) lamps are designed to sanitize the air, objects, and surfaces. However, the prevailing concern is that these UV lamps are often introduced into the market without adequate accompanying information to ensure their safe utilization. Importantly, exposure to absorbed UV light can potentially trigger adverse biological responses, encompassing cell death and senescence.

Our research encompassed a series of investigations aimed at comprehending the biological repercussions of UV-C radiation exposure from readily available domestic lamps. Our focus centered on epithelial retinal cells, keratinocytes, and fibroblasts, components of the skin and ocular targets frequently exposed to UV irradiation.

Our findings underscore the potential harm associated with even brief exposure to UV, leading to irreversible and detrimental alterations in both skin cells and retinal cells of the eye. Notably, epithelial retinal cells exhibited heightened sensitivity, marked by substantial apoptosis. In contrast, keratinocytes demonstrated resilience to apoptosis even at elevated UV doses, though they were prone to senescence. Meanwhile, fibroblasts displayed a gradual amplification of both senescence and apoptosis as radiation doses escalated.

In summary, despite the potential benefits offered by UV-C in deactivating pathogens like SARS-CoV-2, it remains evident that the concurrent risks posed by UV-C to human health cannot be ignored.

## INTRODUCTION

The fight against the spread of the COVID-19 pandemic has caused a state of high alert in global health and the consequent proliferation of various types of sanitization systems [1, 2]. As a result, in a free-market environment, there has been an increase in sales of germicidal lamps using ultraviolet (UV) rays for domestic use, to ensure the sanitization of air, objects, and surfaces. These lights emitting diode (LED) lamps utilize ultraviolet radiation, particularly UV-C (wavelength ranging from 280 nm to 100 nm), which is recognized for its effective germicidal action against viruses, bacteria, spores, and fungi [3]. However, these UV lamps are often not accompanied by sufficient information to ensure their safe use. Furthermore, the individuals using these lamps are often untrained and uninformed [4].

Ultraviolet light is absorbed by a carbon-carbon double bond C=C in thymine and cytosine, two nitrogenous bases of DNA. When this double bond absorbs ultraviolet radiation, it breaks and can react with the nearby nitrogenous base. If this base is another thymine or cytosine, two covalent bonds can form between the two bases. Among the DNA damage induced by UV rays, thymine dimers and the 6–4 photoproduct are worth mentioning [5]. These dimers are dangerous and create a rigid bend in the DNA, causing problems when the cell needs to duplicate its DNA [6].

The International Agency for Research on Cancer (IARC) classifies ultraviolet radiation, including all its components UV-A (400 nm – 315 nm), UV-B (315 nm – 280 nm), and UV-C (280 nm – 100 nm), as Group 1 [7]. In fact, exposure to UV rays causing sunburn has been shown to play a significant role in the development of melanoma, a dangerous form of skin cancer. Additionally, several scientific studies have demonstrated that UV rays can alter tumor suppressor genes, increasing the risk of damaged cells turning into skin cancer. This occurs because UV rays are easily absorbed by nucleic acids, proteins, lipids, and other molecules present within cells [8]. While most of these radiations get absorbed, the remaining portion can alter molecules at a structural level. The damaged molecules, in turn, can react with other molecules inside the cell. Among the documented cellular consequences following UV ray exposure are: point mutations in DNA, DNA damage, denaturation of proteins, apoptosis, and cellular senescence [9, 10]. It should be underlined that UV exposure, besides their contribution to skin cancer, may play a great role in aging phenomena including cellular senescence [11–13].

The senescence process is a complex set of phenomena characterized by a series of molecular and cellular changes that inevitably reflect on the health of organs and entire systems [14]. Senescent cells display various characteristics, including a larger and flattened shape, the presence of senescence-associated β-galactosidase activity, the formation of senescence-associated heterochromatin foci, changes in gene expression, the emergence of telomere-dysfunction-induced foci and the development of senescence-associated secretory phenotype (SASP) [15, 16].

Within our body, senescent cells have dual roles: on one hand, they contribute to the aging process of the entire organism and also participate in tissue development and wound healing. Additionally, these cells can have contrasting effects by acting as inhibitors of cancer or facilitators of tumor growth, depending on the context. Senescent cells execute their diverse functions mainly by releasing the SASP, which consists of various molecules acting as autocrine factors. Notably, components of the SASP also exert their effects as paracrine or long-distance factors, meaning they can influence neighboring healthy cells or even cells distant from the original source of harmful stimuli [15, 17].

For this reason, we conducted a series of studies to evaluate the biological effects of exposure to UV-C radiation from two easily purchasable domestic lamps available on the internet. The lamps in question are: Purple Dawn lamp (referred to as PD) and HVC2654025-16W lamp (referred to as HVC). Both lamps emit spectra centered at a wavelength of 278 nm.

Direct exposure to UV-C radiation can be harmful to both the eyes and the skin. Accidental exposure to UV-C generated by germicidal lamps within the wavelength range of 280 nm to 100 nm can cause serious damage, such as irritations, erythema, burns, severe forms of photokeratitis, retinal damage, and inflammation of the cornea, even with brief exposure [4, 18, 19].

These considerations, together with the before reported UV effects on skin, prompted us to conduct investigations using three different cell lines: human dermal fibroblasts (HDF), spontaneously immortalized human keratinocytes isolated from the epidermis (HACAT), and retinal pigment epithelial cells (ARPE-19).

**Figure 1:**
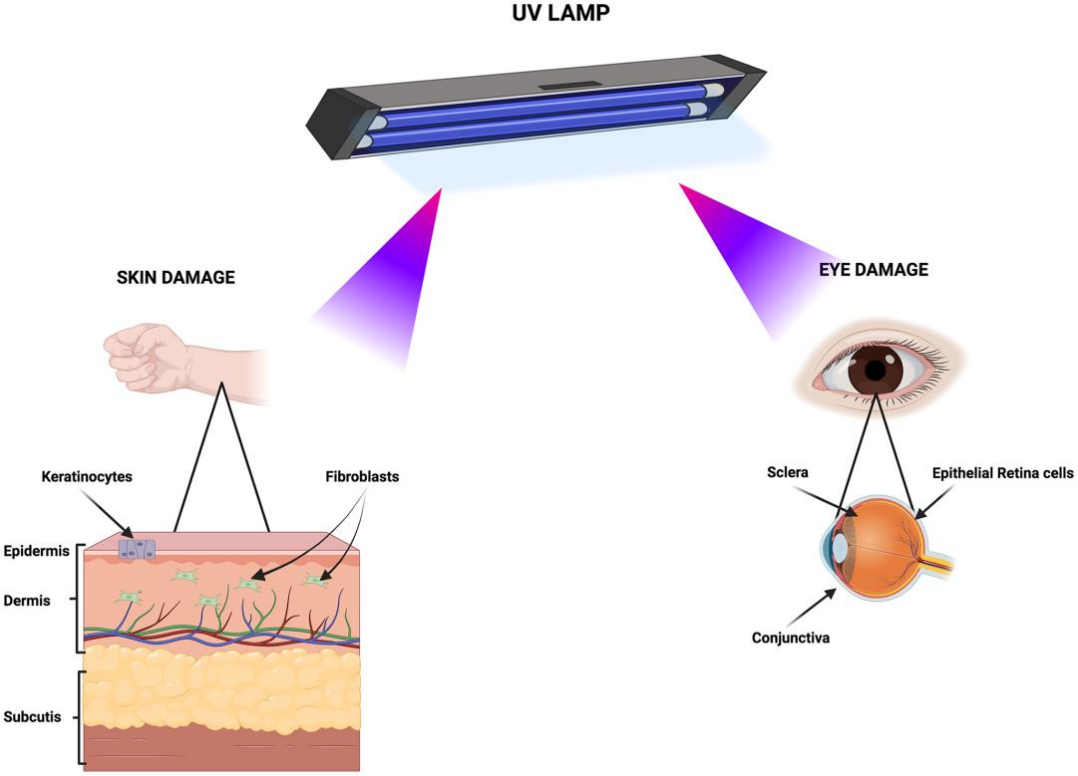
**Primary biological targets of UV exposure.** The cartoon depicts the tissues and cell types susceptible to damage from UV lamp irradiation. Created with BioRender.

These three cell lines were chosen as target model of UV effects on skin and eyes. The skin contains three layers: epidermis, dermis and hypodermis. The keratinocytes and fibroblasts, which are components of epidermis and dermis, respectively, are the cell types most exposed to UV irradiation [20] (Figure 1). The epithelial retinal cells are part of eye’s retina, while fibroblasts are components of sclera and conjunctiva [21]. For their anatomic position these cells are among the main target of eye UV irradiation (Figure 1).

It is known that UV radiation can induce senescence or apoptosis through a direct action, meaning it directly affects the DNA double helix, causing modifications [11, 22–24]. Therefore, our attention was focused on verifying the appearance of phenomena such as senescence, apoptosis, and cellular necrosis in the selected cellular models [17, 25].

## RESULTS

**Figure 2:**
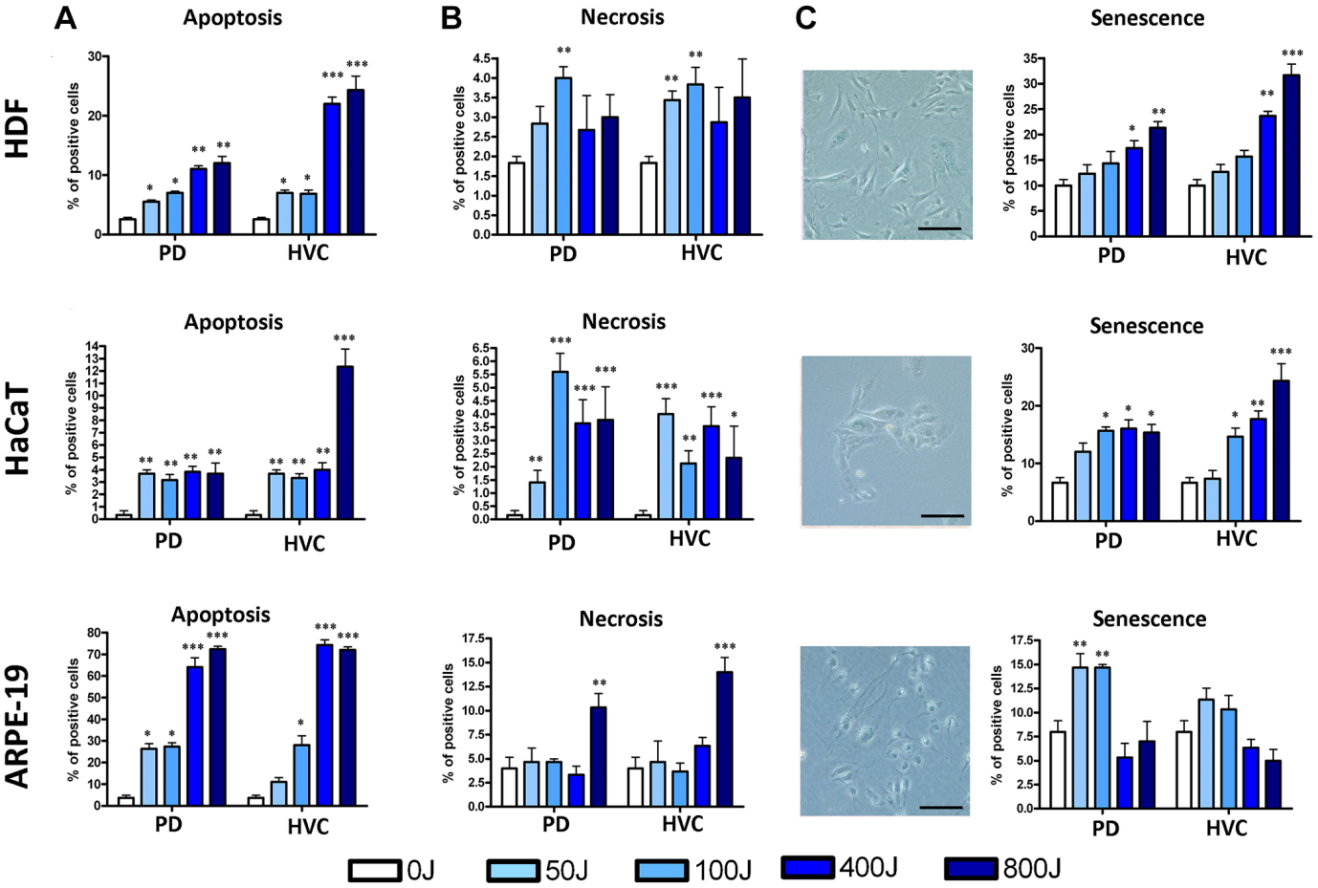
**Biological properties of HDF, HaCaT, and ARPE-19 after UV exposure with two different lamp sources: Purple Dawn (PD) and HVC2654025-16W (HVC).** (**A**) The histograms show the percentage of apoptotic cells identified by the Annexin V assay. This staining enabled the classification of cells into four populations: early and late apoptotic cells, live cells, and necrotic cells. In the graph, early and late apoptotic cells were combined as a single group (*n* = three ± SD) ^*^p < 0.05; ^**^p < 0.01; ^***^p < 0.001. (**B**) The histograms show the percentage of necrotic cells identified by the Annexin V assay (*n* = three ± SD). ^*^p < 0.05; ^**^p < 0.01; ^***^p < 0.001. (**C**) The pictures show representative β-galactosidase staining assay (size bar corresponds to 100 μm). Meanwhile, the histograms show the percentage of senescent cells determined by counting the cells that appear in blue (*n* = three ± SD). ^*^p < 0.05; ^**^p < 0.01; ^***^p < 0.001.

In Figure 2, we present schematic representations of the data obtained from HDF, HaCaT, and ARPE-19 cells irradiated with increasing UV doses using the two selected types of lamps (PD and HVC). The histograms display the percentages of apoptotic cells (early apoptotic: annexin V+ PI- and late apoptotic: annexin V+ PI+), necrotic cells (annexin V− PI+), and senescent cells (identified through the assay of acidic Beta Galactosidase). The results clearly demonstrate that even brief exposure to UV-C radiation is sufficient to induce apoptotic, necrotic, and senescent states in all examined cell types (HDF, HaCaT, ARPE-19). However, each cell type exhibited a distinct biological response pattern to UV irradiation.

ARPE-19 retinal cells displayed heightened sensitivity to UV radiation, showing an increase in apoptosis compared to the control group even at the lowest UV dose. This increase became even more pronounced, reaching up to 70% of apoptotic cells at 800J (Figure 2A). This was also associated with an elevation in necrotic cells (Figure 2B). At the lowest UV dose, ARPE-19 cells also exhibited an increase in senescent cells (Figure 2C).

HaCaT keratinocytes exhibited the highest level of stress resistance among the cell types. We observed an increase in apoptosis even at low doses, yet the percentage of apoptotic cells numerically remained around 4%, notably lower than the percentages observed in other cell lines. An increase in necrosis was also detected compared to unirradiated cells; however, in this case as well, the percentage remained within the range of 4–6% (Figure 2A, 2B). Interestingly, at high doses, a notable percentage of senescent cells was detected (Figure 2C).

HDF fibroblasts demonstrated a progressive rise in apoptosis and senescence as the dose quantity increased (Figure 2A, 2C), although necrosis did not follow the same trend (Figure 2B).

We used two lamps with different watt power and hence the dose rate was different. For example, to administer cells 800Joule UV we irradiated sample for 1200’’ with PD and 480’’ with HVC, respectively. The results observed from both lamps yield comparable biological outcomes, yet it’s crucial to highlight that, on the whole, the intensity of the observed effects tends to be greater when exposed to the HVC lamp (Figure 2A–2C).

**Figure 3:**
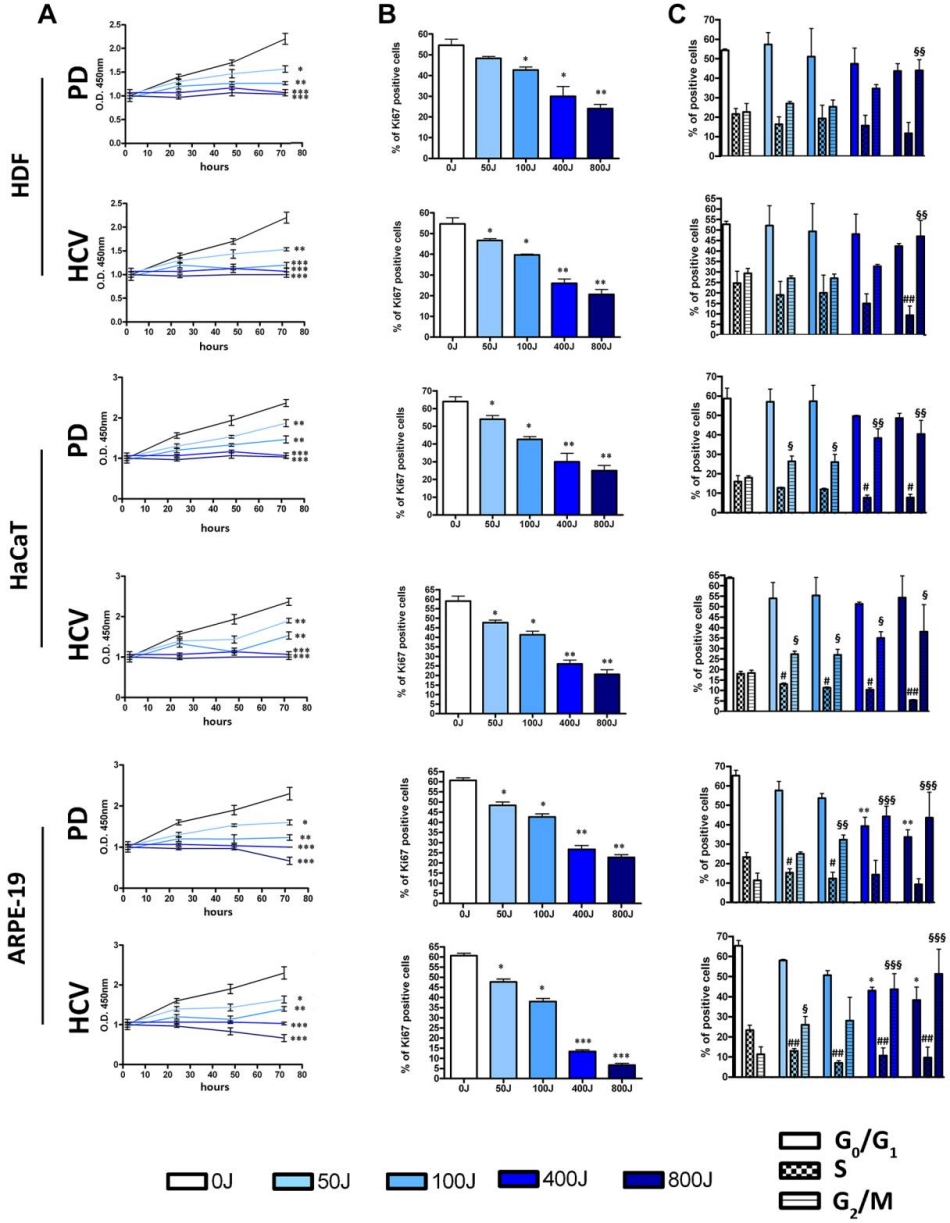
**Effects of UV exposure with two different lamp sources: Purple Dawn (PD) and HVC2654025-16W (HVC) on proliferation of HDF, HaCaT, and ARPE-19.** (**A**) The graphs show the cell proliferation measured by Cell Counting Kit-8 at 24, 48, and 72 h after UV exposure. The graphs display the mean values ± SD (*n* = three). ^*^p < 0.05; ^**^p < 0.01; ^***^p < 0.001. (**B**) The histograms show the percentage of Ki67 positive cells identified by ICC assay (*n* = three ± SD). ^*^p < 0.05; ^**^p < 0.01; ^***^p < 0.001. (**C**) The picture shows the percentages of different cell populations (G1, S, and G2/M) as indicated 72 h after UV exposure. Data are expressed with standard deviation. Experiments were conducted in triplicate for each condition (*n* = three). The ^*^ symbol indicates a comparison among the G1 phase under different experimental conditions (^*^p < 0.05; ^**^p < 0.01), while the ^#^ and ^§^ symbols correspond to the S phase and G2/M phase, respectively (^#^*p* < 0.05; ^##^*p* < 0.01; ^§^*p* < 0.05; ^§§^*p* < 0.01; ^§§§^*p* < 0.001).

The data related to senescence and cell death are further supported by those obtained regarding cell proliferation (Figure 3A). In particular, through the CCK-8 assay performed, a reduction in the percentage of proliferating cells is observed. This is also supported by a decrease in cells positive for the proliferation marker Ki67 (Figure 3B), indicating that following irradiation an increasing number of cells exited cell cycle, irrespective of the final destiny.

Data concerning the cell cycle profiles of irradiated cells are of significant interest. A widely accepted notion is that cells predominantly undergo apoptosis during the G1/S or G2/M phase of the cell cycle [26, 27]. Conversely, the process of senescence has long been associated with a G1 arrest. Nonetheless, within our experimental context, regardless of whether the primary outcome was senescence or apoptosis, we consistently observed an increased presence of cells in the G2/M phase (Figure 3C). This observation suggests that exposure to UV radiation induces a G2/M arrest rather than a G1 block. Furthermore, we also identified a reduction in the number of cells within the S phase, and this decrease achieved statistical significance at higher UV doses (Figure 3C).

Following a genotoxic stress, cells arrest cell cycle and try to repair DNA damage and injury to other types of cellular macromolecules. There are several outcomes of DNA repair process. In some cases, cells completely recover from stress, while in others they can enter apoptosis or senescence. The p53 and retinoblastoma pathways are at the top of hierarchical signaling circuits governing such phenomena [11, 28–31]. Therefore, an analysis of the main players of the two described pathways was performed.

**Figure 4:**
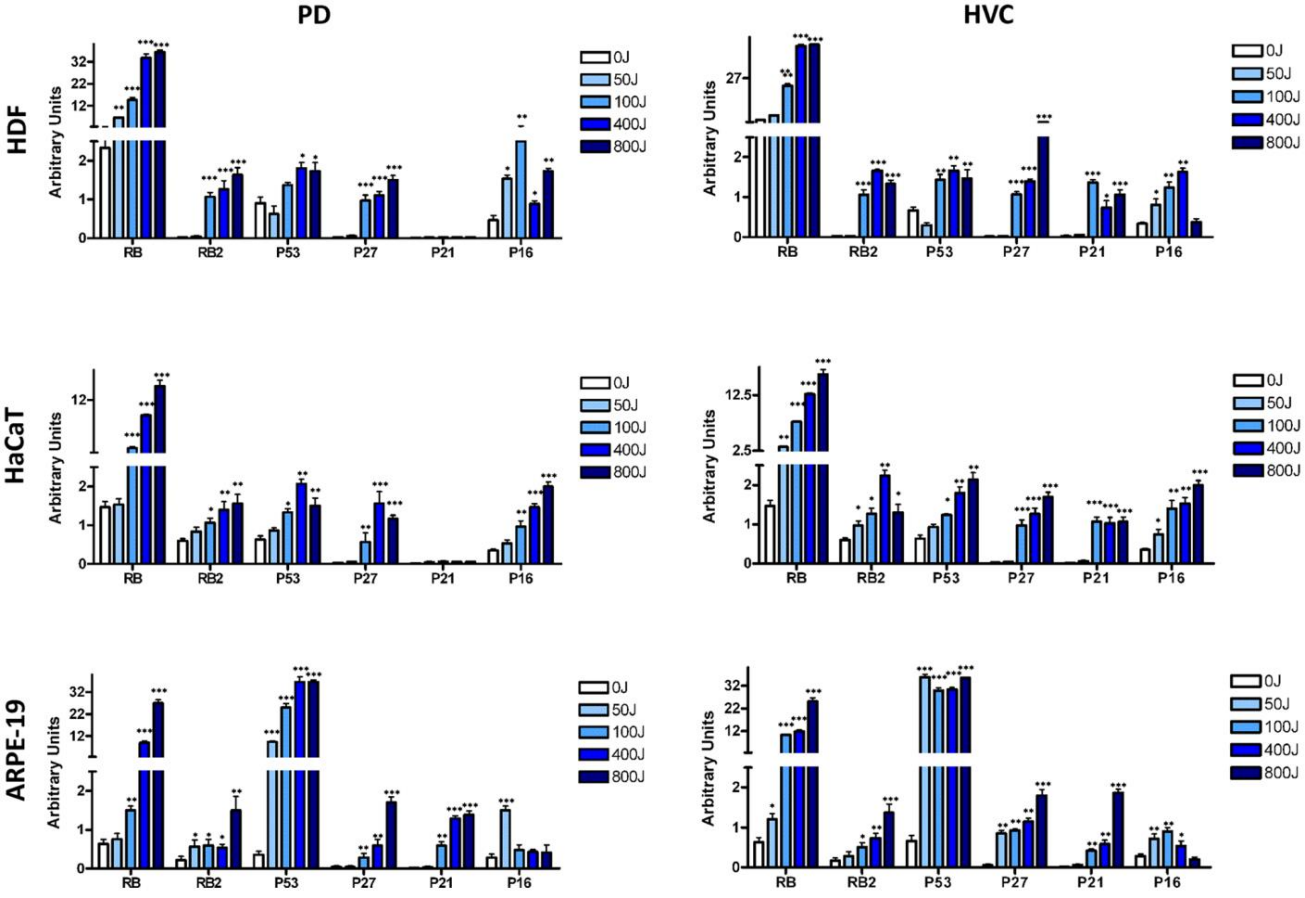
**Analysis of principal pathways involved in senescence, apoptosis, and proliferation by western blot.** The histogram shows the quantitative evaluation of western blot bands in HDF, HaCaT, and ARPE-19 after UV exposure with two different lamp sources: Purple Dawn (PD) and HVC2654025-16W (HVC) as indicated. The data are expressed as Arbitrary Units (A.U.) with the mean expression values (± SD, *n* = three). ^*^p < 0.05; ^**^p < 0.01; ^***^p < 0.001.

In all three different cell lines, we observed an increase in RB, with the most notable elevation seen in HDF cells (Figure 4 and Supplementary File 1). The upregulation of P53 was predominantly noted in ARPE-19 cells, exhibiting a consistently significant increase in expression following UV exposure. However, in HFD and HaCaT cells, we only observed a moderate upregulation of P53 (Figure 4).

The upregulation of P21CDKN1A and P16INK4A, which play crucial roles in apoptosis and senescence, respectively, followed the overall biological trend observed in each cell line. Specifically, ARPE-19 cells, which were more prone to apoptosis, especially at high UV doses, demonstrated a progressive increase in P21CDKN1A. Additionally, P16INK4A expression was higher at 50J irradiation, coinciding with an observed increase in senescence (Figure 4).

In the other two cell lines, the increase in P21CDKN1A was modest, consistent with low levels of apoptosis, while the upregulation of P16INK4 generally followed the onset of senescence (Figure 4). The expression levels of RB2/P130 and P27CDKN1B play a general role in governing a permanent cell cycle exit [32] and were progressively upregulated in all the analyzed cell lines (Figure 4).

## DISCUSSION

Highlighting the increasing popularity of germicidal lamps that utilize UV-C radiation for surface disinfection, despite the well-known risks associated with UV exposure. The availability of these devices on the internet raises concerns due to the lack of proper knowledge among users, potentially leading to unsafe usage.

The report’s data indicates that even brief exposure to UV-C radiation from these lamps (as little as 70 seconds for the Purple Dawn lamp and 30 seconds for the HVC2654025-16W lamp) can cause irreversible and harmful changes in both skin cells and retinal cells of the eye.

Biological outcomes obtained with the two lamps are similar, although it must be emphasized that, in general, the intensity of observed phenomena is higher with HVC lamp exposure. For instance, the percentage of apoptotic cells in HDF cells exposed to 400 and 800J UV is higher with HVC treatment compared to PD irradiation. This difference may be attributed to the dose/rate, which is higher in HVC compared to PD. In certain settings, cells can better tolerate a given quantity of radiation administered at low dose rates compared to the same quantity given in short time periods [33]. This result raises significant safety concerns about the use of germicidal lamps, as companies in this field are concentrating their efforts on producing lamps that will deliver a germicidal effect in a very short time to align with the preferences of public opinion.

The sensitivity to UV stress varies among the three cell lines, with retinal cells being the most sensitive, while keratinocytes are more resistant to injury. The retinal cells underwent massive apoptosis at the highest UV dose, while they entered senescence at lower doses. Reports suggest that, in certain cellular contexts, the cell’s choice between apoptosis and senescence following a DNA damage event depends on the intensity of the damage [31]. This seems to be the case for retinal cells, as at the highest doses, a fraction of cells become necrotic. Necrosis is a passive cell death that occurs when the intensity of DNA damage does not allow for the activation of any active cellular stress response.

The presence of massive apoptosis phenomena in ARPE-19 cells is associated with strong increment of P53-P21CDKN1A pathway, which governs cell death process.

The keratinocytes exhibited minimal apoptosis levels even at the highest UV doses, yet they displayed a susceptibility to senescence. This aligns with the adaptation of keratinocytes to the presence of UV rays in sunlight. These cells have developed defense mechanisms to effectively manage the DNA damage prompted by UV exposure, potentially relying on the functional state of the insulin-like growth factor-1 receptor (IGF-1R) signaling network. It has been suggested that pathways associated with IGFs, when activated, play a pivotal role in safeguarding keratinocytes against UV-induced apoptosis. Nevertheless, a repercussion of this protective mechanism is the dampening of cell proliferation through the initiation of senescence [34]. Indeed, in these cells we detected an activation of the RB-P16INK4A pathway.

The fibroblasts exhibited a distinct response pattern to UV irradiation, marked by a gradual escalation of both senescence and apoptosis as radiation doses increased. While necrosis did show an elevation in irradiated cells when compared with controls, no clear trend was discernible with respect to dose quantity. This outcome confirms that the straightforward correlation of senescence, apoptosis, and necrosis with the intensity of DNA damage may hold true only under certain circumstances, as seen in the case of retinal epithelial cells. This result underscores the intricate nature of studying cellular responses to DNA damage, posing a considerable challenge. To narrow the scope of hypotheses within the framework of our experimental model, we could conjecture that fibroblast cultures may exhibit greater heterogeneity than epithelial retinal cultures, potentially comprising various subpopulations with differing capacities to manage DNA damage.

The conventional understanding is that cells typically undergo apoptosis during the G1/S or G2/M phase of the cell cycle [26, 27], whereas the senescence process has been traditionally associated with a G1 arrest. However, recent reports have introduced the idea that senescence can also manifest with a G2/M block [35, 36]. In our experimental model, regardless of the primary outcome—whether senescence or apoptosis—we observed an elevated presence of cells in the G2/M phase, a presence that was notably higher compared to the control samples when subjected to high UV doses. This finding indicates that UV exposure induces a G2/M arrest rather than a G1 block. It is plausible to speculate that during the S phase, DNA becomes more susceptible to UV irradiation due to its single-stranded nature and its lack of nucleosome protection during the replication process. Consequently, this vulnerability could lead to the accumulation of DNA damage, ultimately triggering the G2/M checkpoint to halt the progression of the cell cycle.

The P53 and retinoblastoma pathways play critical roles in regulating cell cycle arrest, DNA damage response, apoptosis, and senescence. In our experimental model, we did not observe a consistent response to UV irradiation, as both senescence and apoptosis were triggered by UV treatment. The distinction lay in the prevalence of one phenomenon over the other in specific circumstances. For instance, high doses of UV primarily induced apoptosis in retinal epithelial cells. Within this context, it remains challenging to ascertain which specific P53/retinoblastoma circuit governed the observed biological occurrences. Further investigation, employing RNA single-cell analysis, could potentially offer clarity on this matter. Our data, however, may suggest specific trends: in ARPE-19, the biological effects are mainly governed by the P53-P21CDKN1A pathway, while in HaCaT, they are regulated by the RB-P16INK4A pathway.

## CONCLUSIONS

Despite the potential advantages of utilizing UV-C radiation for deactivating pathogens such as SARS-CoV-2, the prevailing conclusion remains that UV-C radiation poses concurrent risks to human health. The data acquired and presented in this report make it apparent that mere seconds of exposure (70 seconds for the Purple Dawn lamp and 30 seconds for the HVC2654025-16W lamp) are sufficient to induce irreversible and detrimental alterations in both skin cells and retinal cells of the eye. This raises apprehensions regarding the safety of employing germicidal lamps for disinfection purposes in the absence of adequate precautions and user awareness. Two primary concerns stand out as particularly perilous for health: the significance of dose rate in determining the severity of adverse biological effects and the heightened vulnerability of retinal epithelial cells to irradiation from these germicidal lamps. Further research and heightened awareness are imperative to ensure the prudent and secure application of such technology.

## METHODS

### Cell cultures

Immortalized keratinocyte cell line (HaCaT), Primary Dermal Fibroblast (HDF) and arising retinal pigment epithelia (RPE) cell (ARPE-19) were obtained from ATCC (VA, USA). The cells were grown according to manufacturers’ instruction. HaCaT cells were cultured in Dulbecco’s modified Eagle’s medium (DMEM) supplemented with 10% fetal bovine serum and 1% antibiotics (10,000 μg/ml streptomycin and 10,000 units/ml penicillin) at 37°C in a 5% CO_2_ environment. The cells underwent sequential passages when reaching a confluence level of 70–80%. The same experimental conditions were applied to ARPE-19 and HDF, with the only distinction being the use of DMEM: F12 medium for ARPE-19 and DMEM low glucose for HDF.

### UV exposure

The cells were subjected to UV exposure at 260 nm using two distinct lamps: Purple Dawn (Sonnenkoning, Switzerland) and HVC2654025-16W (Midland Europe, Italy). These lamps possess varying wattage capacities. Specifically, the Purple Dawn lamp emits at 0.7 W/m2, while the HVC2654025-16W lamp operates at 1.6 W/m2. The cells were irradiated with different energy levels (measured in joules): 0J, 50J, 100J, 400J, and 800J. Consequently, the exposure times differ for each lamp, as illustrated in the schematic below. Both lamps were positioned over Petri dishes containing the cells, without coverslips, at a vertical distance of 5 cm from the lamp to ensure uniform irradiation [37]. Following exposure, the cells were cultured for 72 hours under incubation conditions of 37°C, 5% CO_2_, and controlled humidity.

Schedule of time and power exposure:

**Table:** 

	0J	50J	100J	400J	800J
Purple Dawn lamp	0’’	70’’	140’’	600’’	1200’’
HVC2654025-1.6W lamp	0’’	30’’	60’’	240’’	480’’

### *In situ* senescence-associated beta-galactosidase assay

Following UV exposure, cells were fixed in a solution containing 2% formaldehyde. Subsequently, the cells underwent thorough washing with phosphate-buffered saline (PBS) (Microgem, Italy). They were then incubated at a temperature of 37°C for an overnight period within a staining solution. This solution consisted of citric acid/phosphate buffer (pH 6), K_4_Fe (CN)_6_, K_3_Fe (CN)_6_, NaCl, MgCl_2_, and X-Gal. To assess the proportion of senescent cells, a count was conducted on the cells displaying β-galactosidase-positive characteristics, indicated by their blue coloration. This count was performed across multiple microscopic fields, encompassing no fewer than 300 cells in each instance, adhering to the methodology described in previous work [38]. Unless specifically mentioned, all reagents utilized in this study were procured from Sigma-Aldrich (MO, USA).

### Annexin V assay

We utilized a Guava^®^ easyCyte™ flow cytometer (Millipore-Sigma, MA, USA) for the detection of apoptotic cells, employing fluorescein-conjugated annexin V from an Annexin kit provided by Dojindo Molecular Technologies (MD, USA). This process adhered to the guidelines outlined by the manufacturer. The kit incorporated two distinct dyes, Annexin V and propidium iodide (PI), serving to differentiate between apoptotic and non-apoptotic cells. Annexin V (labeled in green) selectively bound to phosphatidylserine on the outer membrane of apoptotic cells, while PI (appearing in red) permeated the cells and labeled the DNA of those in the late stages of apoptosis or deceased. This dual staining facilitated the categorization of cells into four distinct populations: non-apoptotic cells (annexin V− and PI−); early apoptotic cells (annexin V+ and PI−); late-apoptotic cells (annexin V+ and PI+), and necrotic cells (annexin V− and PI+). For our experimental purposes, early and late apoptotic cells were combined into a single group.

### Cell proliferation

To evaluate the viability of the cells, we employed a colorimetric assay known as the Cell Counting Kit-8 (CCK-8) obtained from Dojindo Molecular Technologies (MD, USA). This assay involves the utilization of a water-soluble tetrazolium salt named WST-8, which is subject to reduction by the cellular dehydrogenase activity, leading to the creation of a yellow-colored formazan dye. The amount of formazan dye generated is directly proportional to the count of viable cells present. We conducted measurements of cell viability at three time points: 24 hours, 48 hours, and 72 hours following exposure to UV radiation. The microplate reader employed for this assay was the Infinite 200 from Tecan (Switzerland), and the readings were taken at a wavelength of 450 nm.

### Cell cycle analysis

The cells were collected and subsequently treated with 70% ethanol at a temperature of −20°C for an overnight duration. Following this, the cells underwent a thorough rinsing process with 1X PBS three times. They were then suspended in a hypotonic buffer containing PI (procured from Sigma-Aldrich). The resulting cell samples were subjected to analysis using a Guava^®^ easyCyte™ flow cytometer, provided by Millipore-Sigma, in accordance with the established protocol outlined by the easyCyte™ software.

### Western blotting

Cell lysis was carried out by treating the cells with a buffer containing 0.1% Triton-X100 (Roche Life Sciences, IN, USA) for a duration of 30 minutes on ice. Each protein lysate was subjected to electrophoresis, with a quantity of twenty micrograms loaded onto a polyacrylamide gel. Subsequently, the proteins were transferred onto a nitrocellulose membrane via electroblotting. The primary antibodies utilized in this study included RB1 and P27CDKN1B from Cell Signaling Technology (MA, USA), RB2/P130 from BD Biosciences (CA, USA), P107, P53 (DOI-1), P21CDKN1A (C-19) from Santa Cruz Biotechnology (CA, USA), and P16INK4A from Abcam (United Kingdom).

In order to detect immunoreactive signals, a secondary antibody conjugated with horseradish peroxidase was employed. This secondary antibody was sourced from Santa Cruz Biotechnology and it was used in conjunction with the ECL plus reagent from GE Healthcare (IL, USA). The application of all antibodies followed the instructions provided by their respective manufacturers.

### Statistical analysis

To assess statistical significance, a series of tests was employed, starting with ANOVA followed by Student’s *t*-test and Bonferroni tests. For data with continuous outcomes, a mixed-model variance analysis was utilized. All data analyses were conducted using GraphPad Prism version 8 statistical software package provided by GraphPad Software (CA, USA).

### Availability of data and materials

The main data supporting the findings of this study are available within the article. Unprocessed western blot images are available upon request.

## Supplementary Materials

Supplementary File 1
