# Supplementary material for: Germicidal lamps using UV-C radiation may pose health safety issues: a biomolecular analysis of their effects on apoptosis and senescence
Source: Aging (Albany NY). 2024 May 2;16(9):7511–22. doi: 10.18632/aging.205787 (PMC11131978; doi:10.18632/aging.205787)
Supplement: Supplementary File 1 [file aging-16-205787-s001.pdf]

Supplemntary File 1. WB Raw data. The file contains the original blots of data showed in Figure 4.

HDF

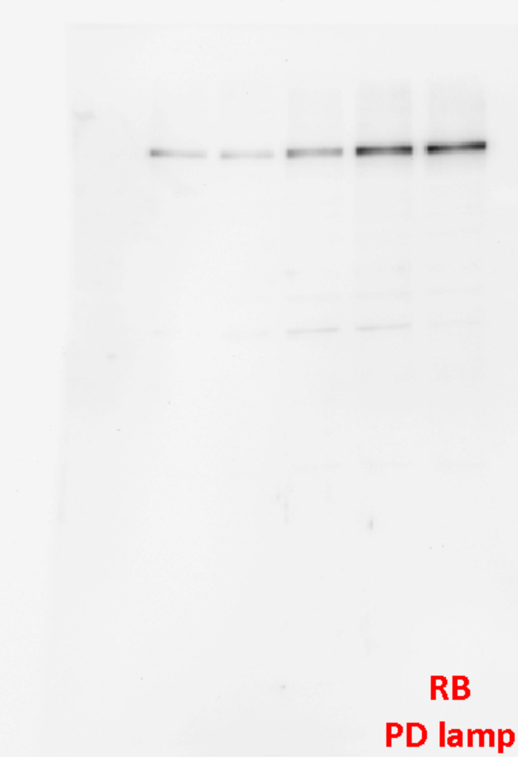

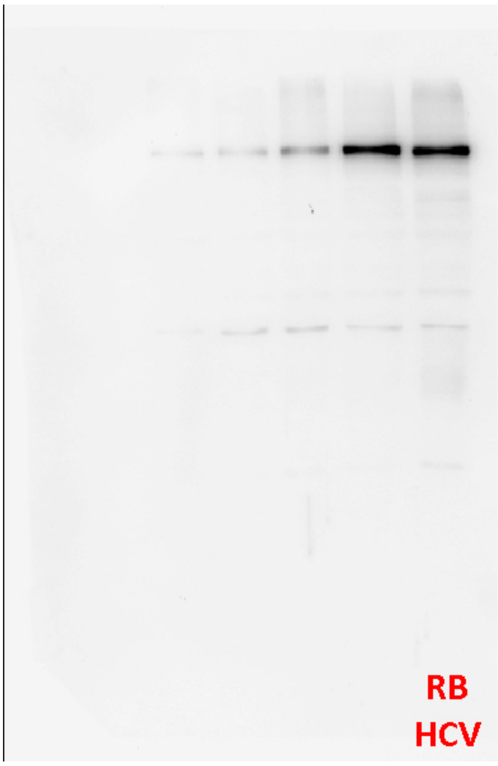

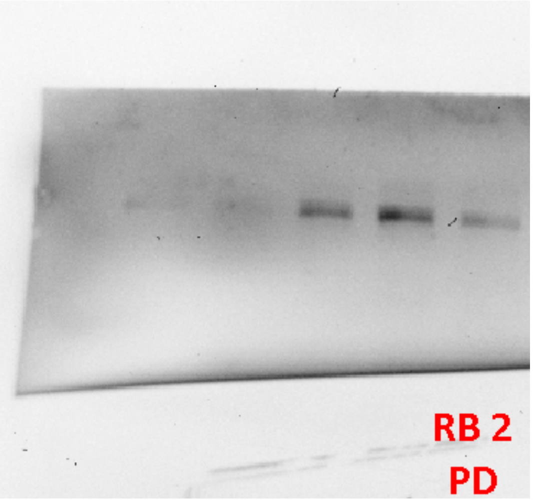

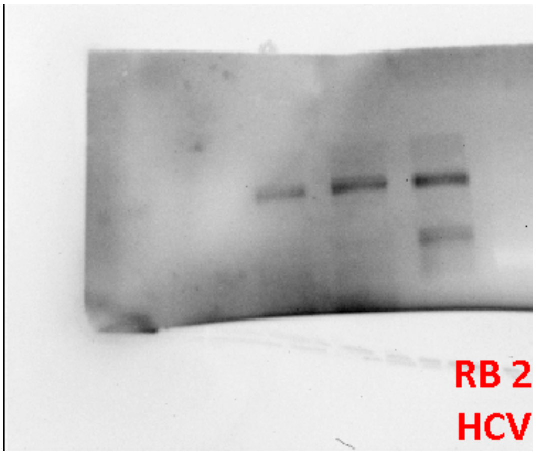

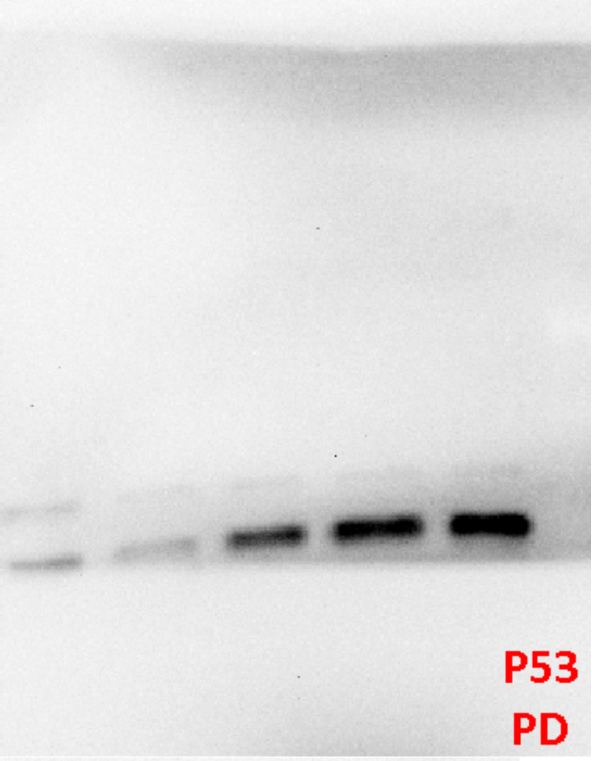
P53
PD

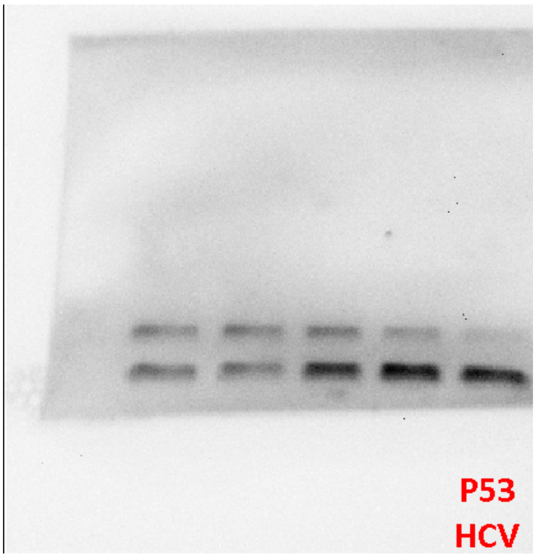
P53
HCV

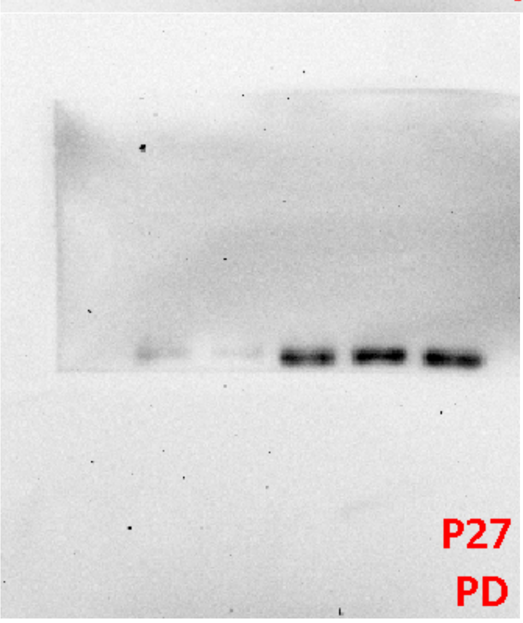
P27
PD

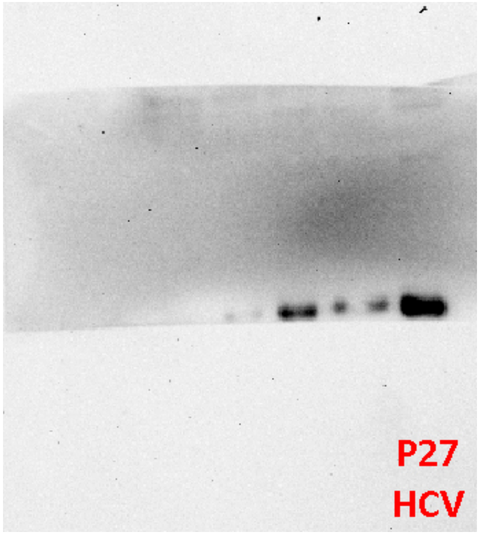
P27
HCV

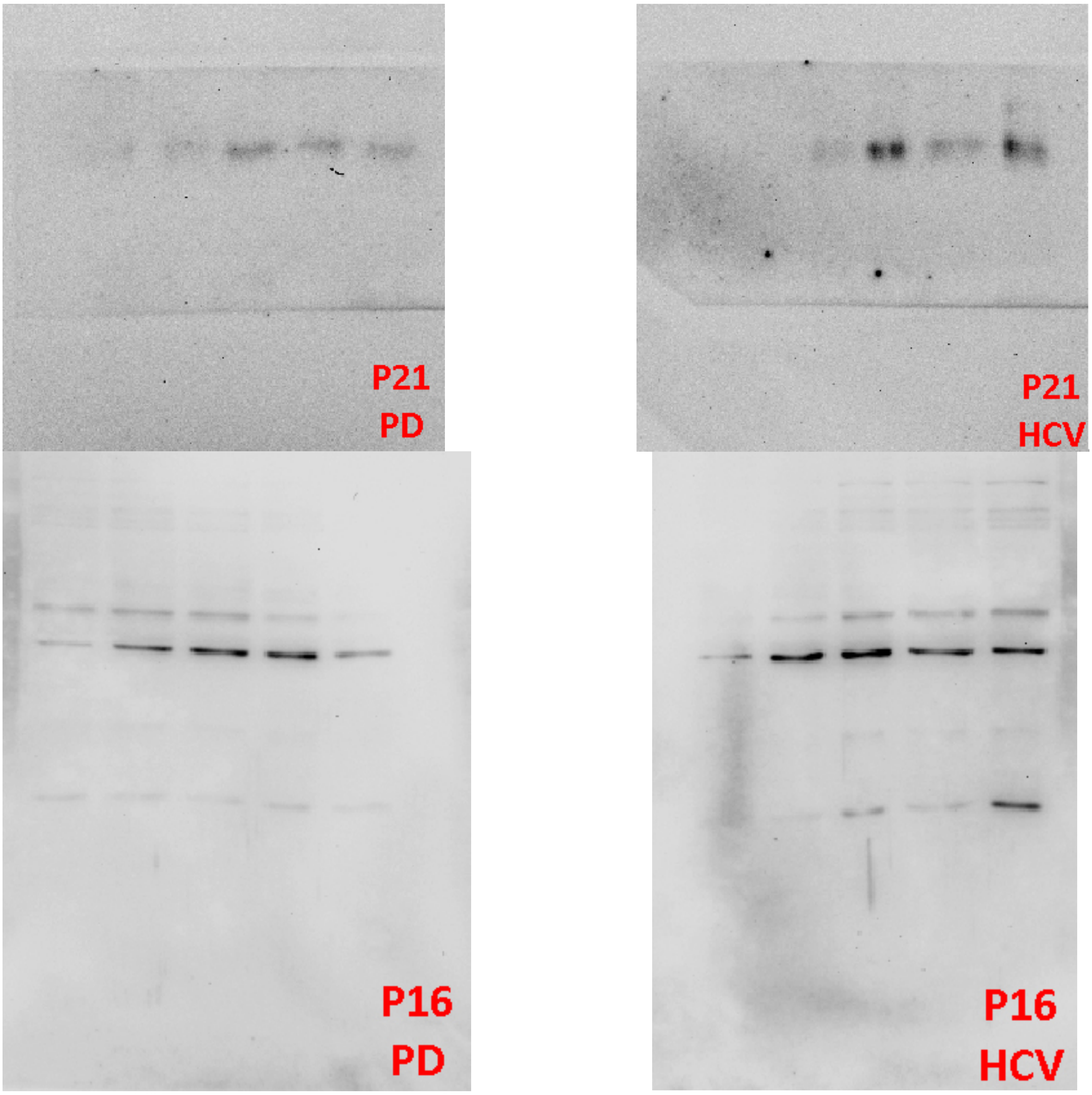
P21
PD
P21
HCV
P16
PD
P16
HCV

HaCaT

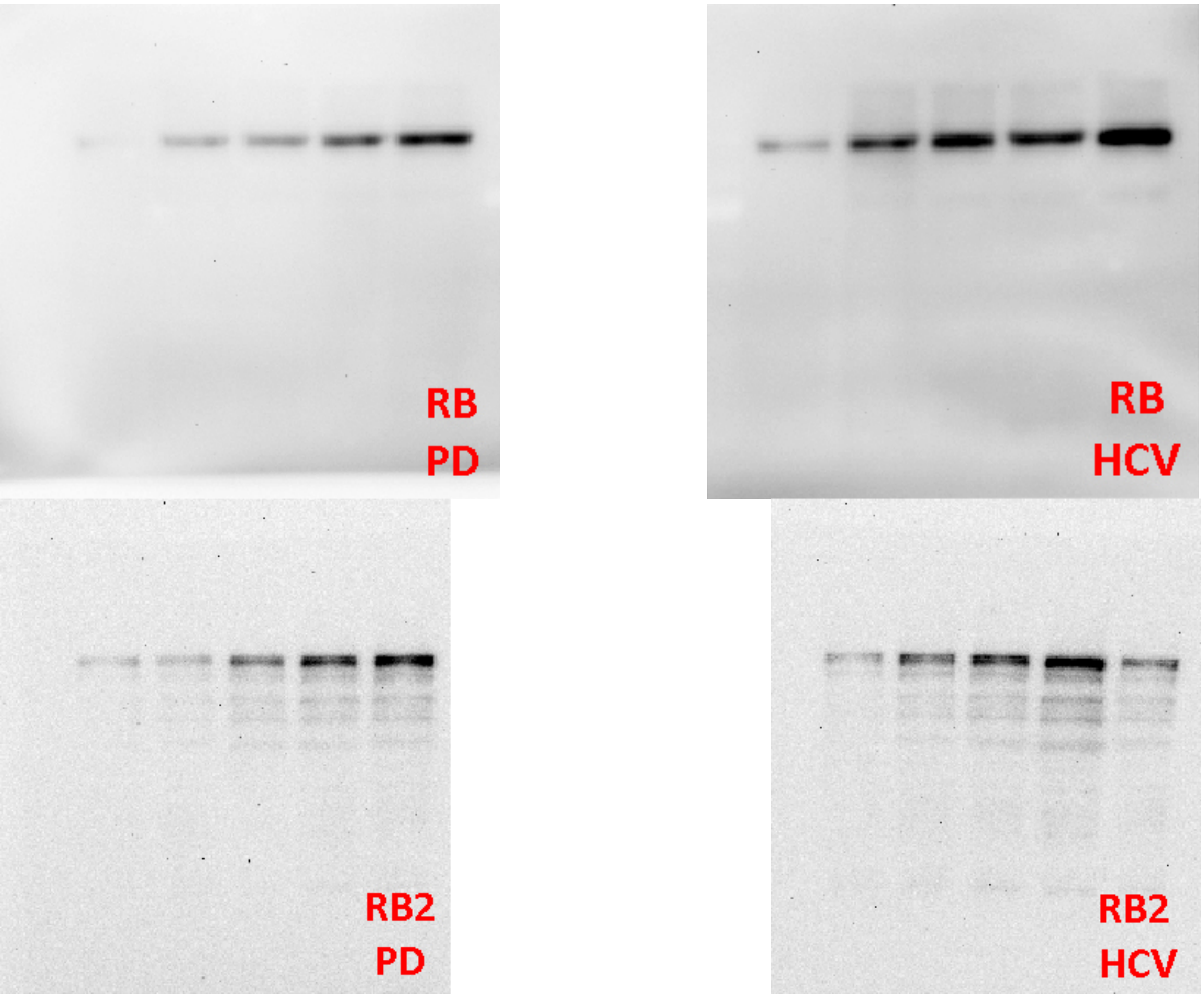

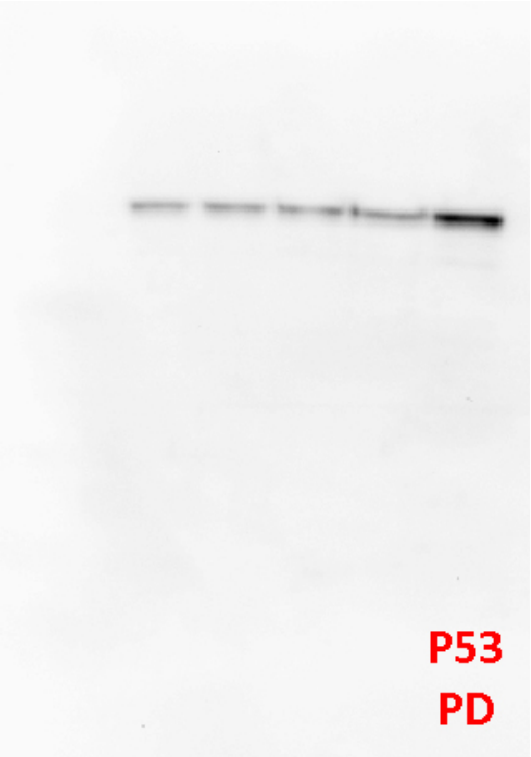
P53
PD

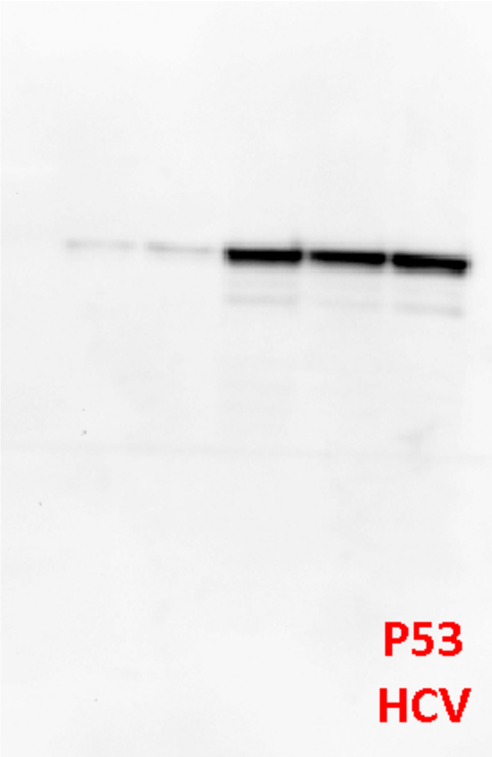
P53
HCV

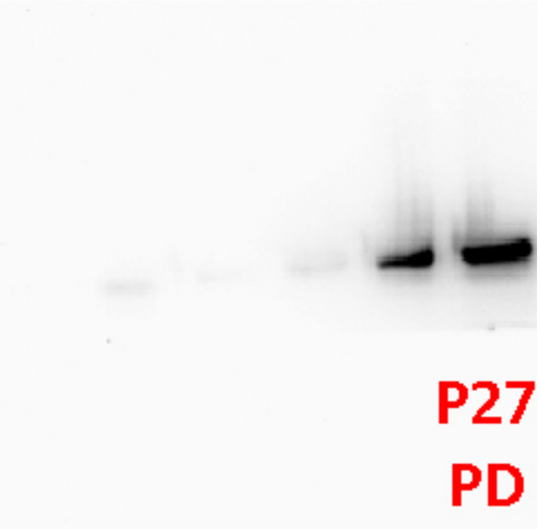
P27
PD

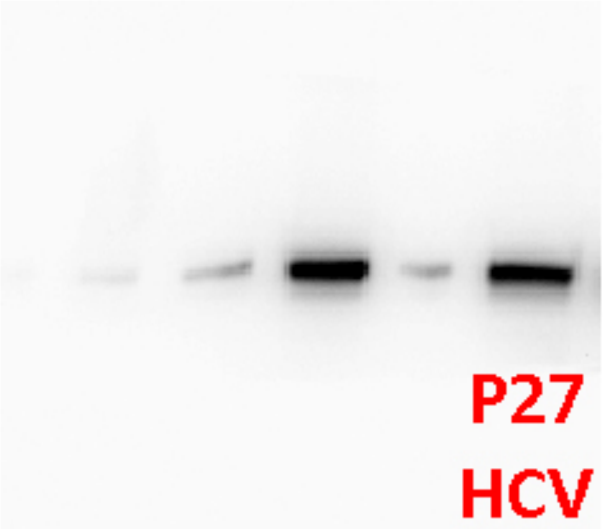
P27
HCV

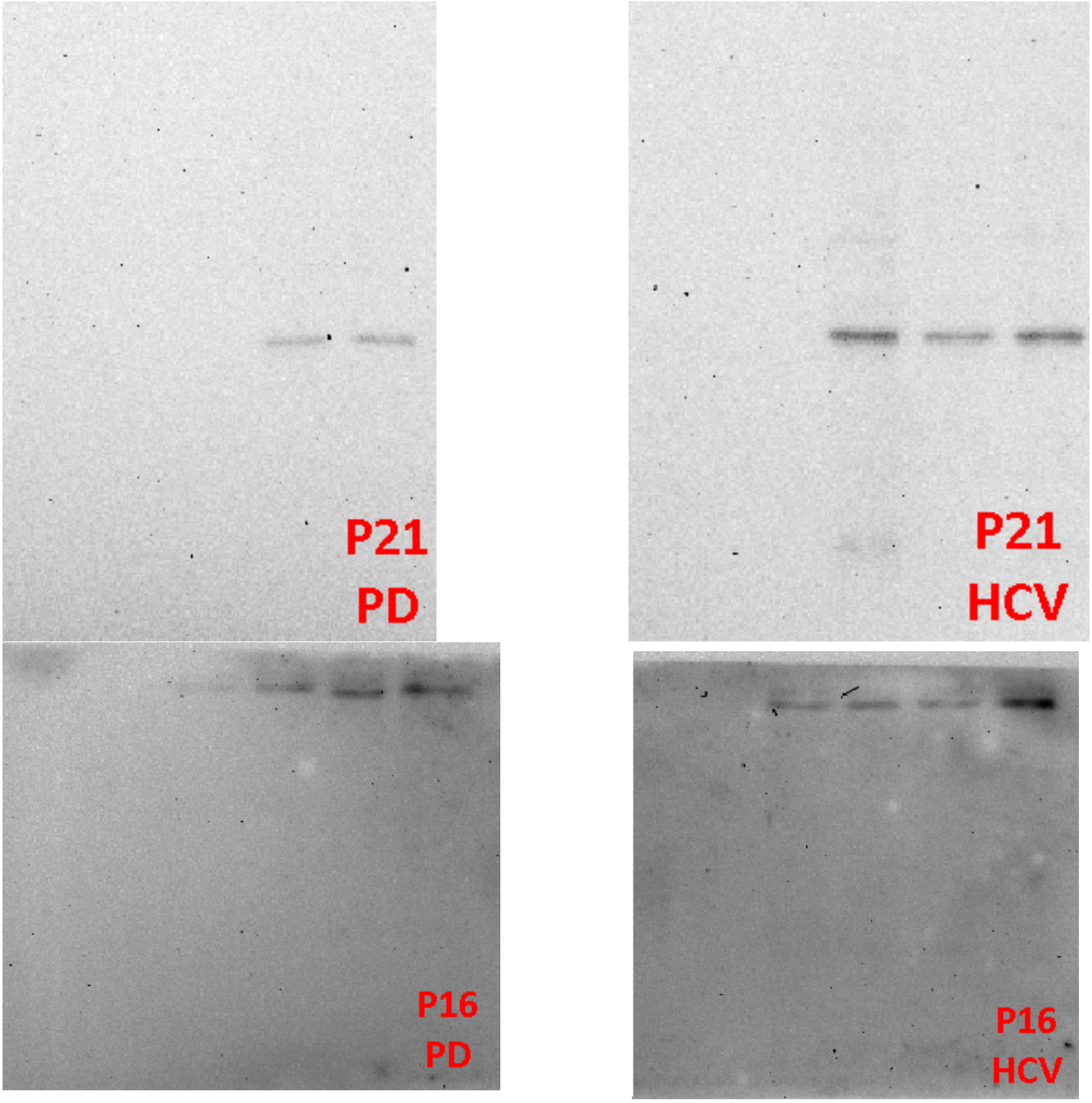
P21
PD
P21
HCV
P16
PD
P16
HCV

ARPE-19

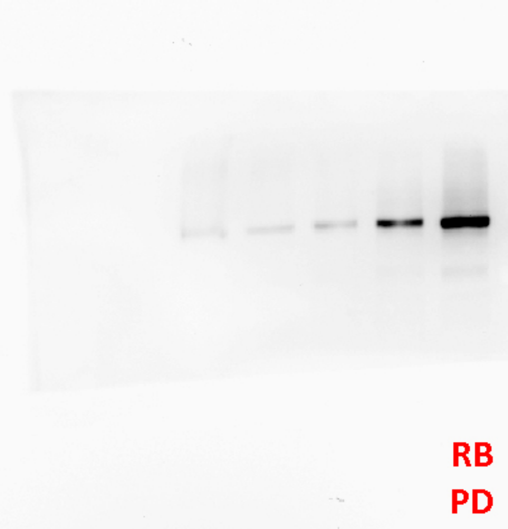

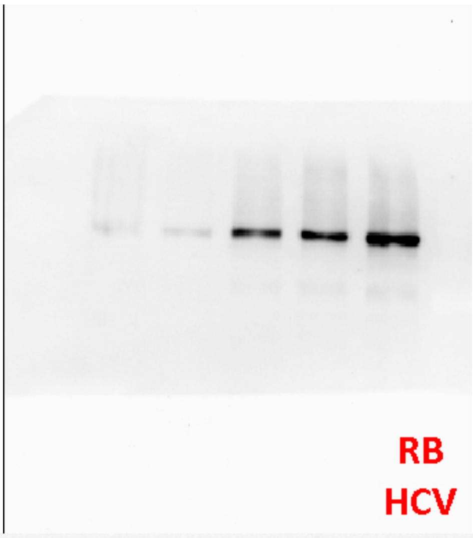

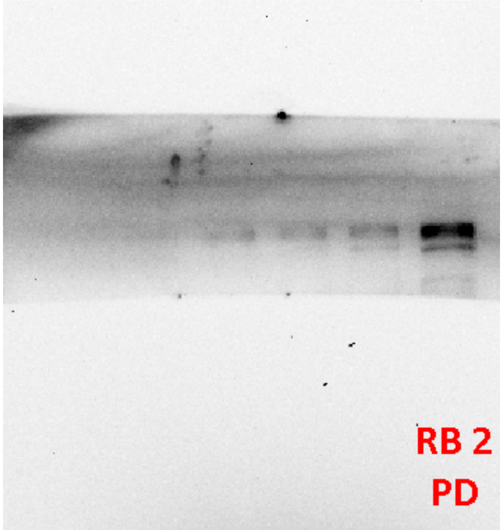

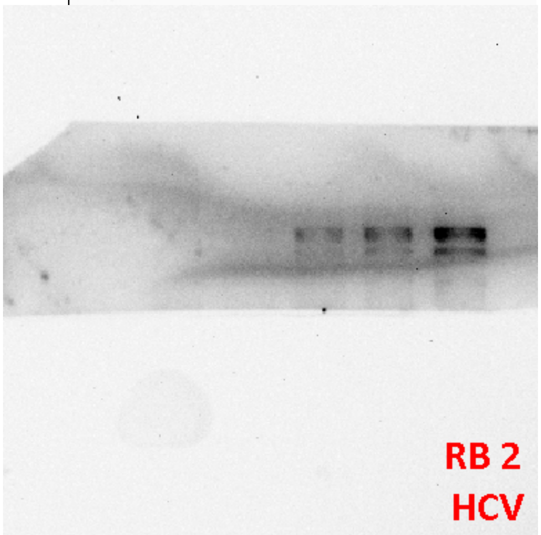

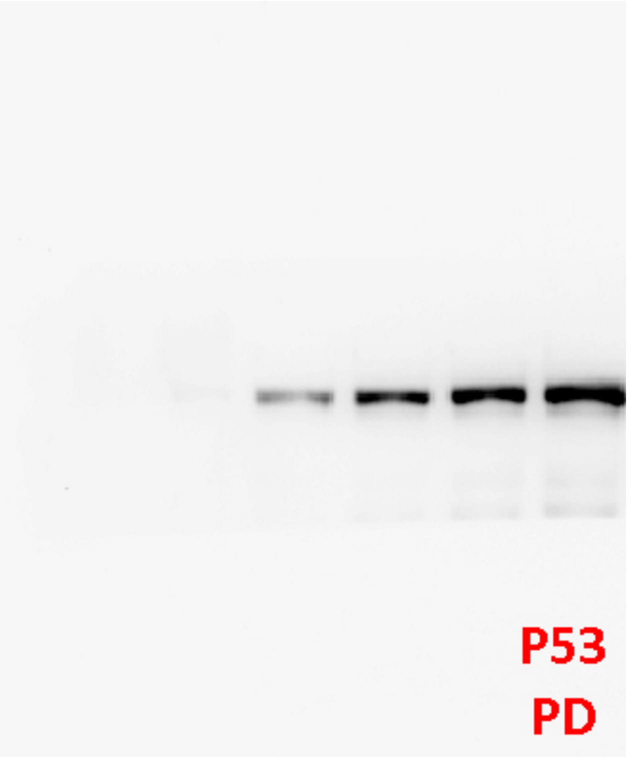
P53
PD

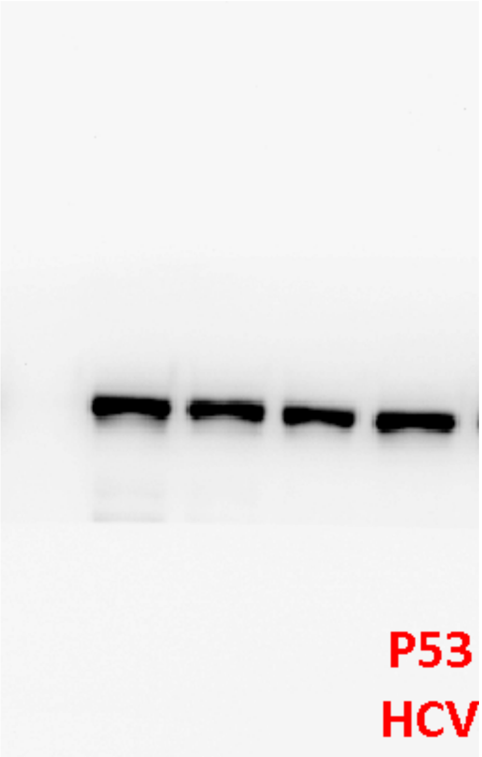
P53
HCV

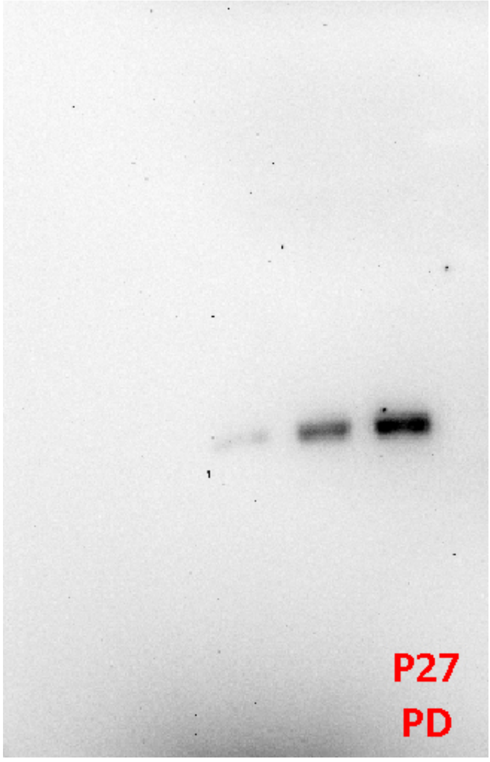
P27
PD

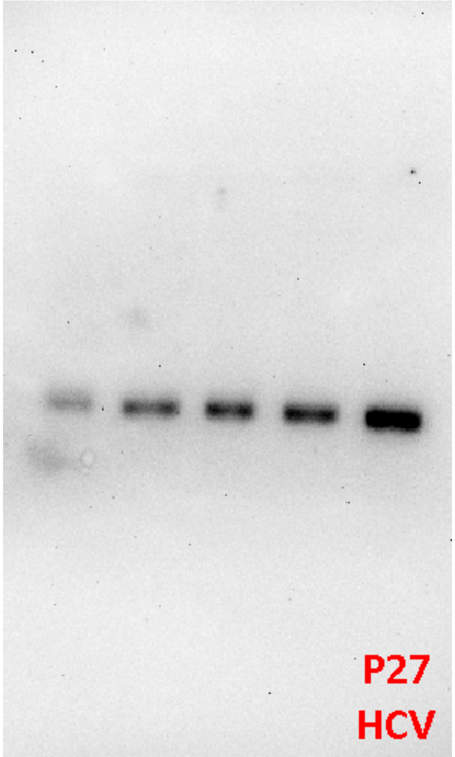
P27
HCV

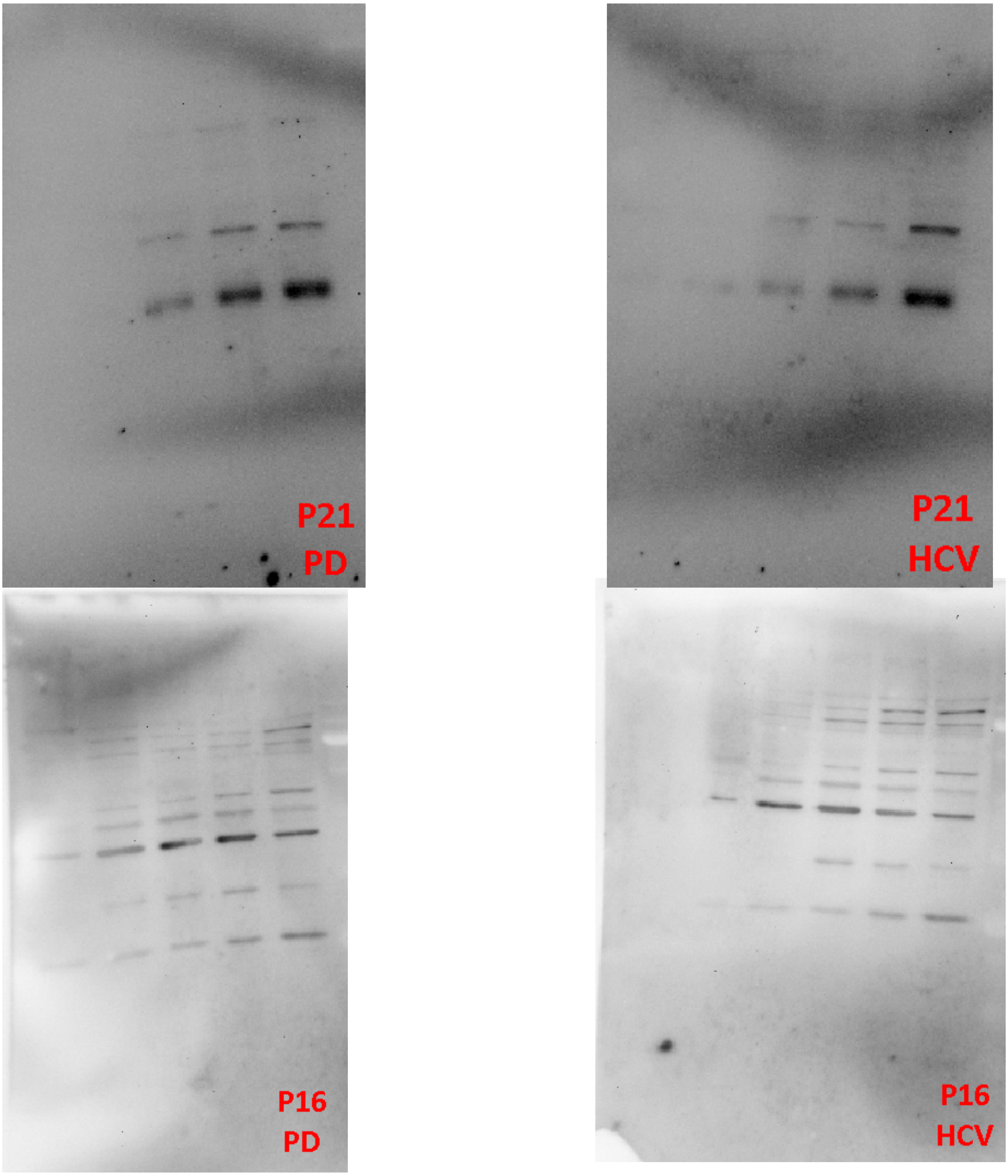
P21
PD
P21
HCV
P16
PD
P16
HCV
